# ATG7 is a haploinsufficient repressor of tumor progression and promoter of metastasis

**DOI:** 10.1073/pnas.2113465119

**Published:** 2022-07-06

**Authors:** Jaclyn S. Long, Elżbieta Kania, David G. McEwan, Valentin J. A. Barthet, Martina Brucoli, Marcus J.G.W. Ladds, Christoph Nössing, Kevin M. Ryan

**Affiliations:** ^a^Tumour Cell Death and Autophagy Laboratory, Cancer Research UK Beatson Institute, Glasgow, G61 1BD, United Kingdom;; ^b^Institute of Cancer Sciences, University of Glasgow, Glasgow, G61 1BD, United Kingdom

**Keywords:** autophagy, ATG7, metastasis, pancreatic cancer

## Abstract

The role of autophagy in cancer is complex. Both tumor-promoting and tumor-suppressive effects are reported, with tumor type, stage and specific genetic lesions dictating the role. This calls for analysis in models that best recapitulate each tumor type, from initiation to metastatic disease, to specifically understand the contribution of autophagy in each context. Here, we report the effects of deleting the essential autophagy gene *Atg7* in a model of pancreatic ductal adenocarcinoma (PDAC), in which mutant *Kras^G12D^* and mutant *Trp53^172H^* are induced in adult tissue leading to metastatic PDAC. This revealed that *Atg7* loss in the presence of *Kras^G12D^*^/+^ and *Trp53^172H^*^/+^ was tumor promoting, similar to previous observations in tumors driven by embryonic *Kras^G12D^*^/+^ and deletion of *Trp53*. However, *Atg7* hemizygosity also enhanced tumor initiation and progression, even though this did not ablate autophagy. Moreover, despite this enhanced progression, fewer *Atg7* hemizygous mice had metastases compared with animals wild type for this allele, indicating that ATG7 is a promoter of metastasis. We show, in addition, that *Atg7*^+/−^ tumors have comparatively lower levels of succinate, and that cells derived from *Atg7*^+/−^ tumors are also less invasive than those from *Atg7*^+/+^ tumors. This effect on invasion can be rescued by ectopic expression of *Atg7* in *Atg7*^+/−^ cells, without affecting the autophagic capacity of the cells, or by treatment with a cell-permeable analog of succinate. These findings therefore show that ATG7 has roles in invasion and metastasis that are not related to the role of the protein in the regulation of autophagy.

## Significance

Many studies investigating the role of autophagy in cancer have involved deletion of *Atg7*. Our finding that *Atg7* hemizygosity affects tumor progression, metastasis, and cellular invasion in an autophagy-independent manner highlights significant diverse roles for ATG7. These results therefore both increase our understanding of the roles of this allele in cancer and indicate that partial inhibition of ATG7 may potentially be a way to treat advanced malignant disease without affecting the beneficial forms of autophagy in normal tissue.

The preservation of cellular integrity and the ability to adapt to different forms of cellular stress are key in the prevention of various forms of disease. Autophagy constitutes a group of processes that facilitates these goals by delivering cellular constituents to lysosomes for degradation and recycling (1). The cargoes destined for degradation can either be targeted specifically to promote cellular health and integrity, or simply digested nonselectively in response to cellular cues such as nutrient deprivation or tissue remodeling (1).

The best characterized form of autophagy is macroautophagy, which is often and hereafter referred to more simply as autophagy. Autophagy involves a number of evolutionarily conserved ATG proteins that orchestrate the formation of a double-membraned structure called an autophagosome which sequesters cellular cargo (2, 3). Autophagosomes then traffic to and fuse with lysosomes to form another structure termed the autolysosome, within which degradation occurs (1). The ability to understand the function of autophagy has been facilitated by the identification that a number of ATG proteins, including ATG7 and ATG5, are essential for autophagosome formation, and mice containing floxed alleles for the genes encoding these proteins have been fundamental in understanding the role of autophagy in various forms of mammalian physiology and disease (2–5).

It is now clear that autophagy has a major role in both preventing and sustaining cancer (6). Several autophagy genes have been shown to be mutated in major forms of cancer, and mice lacking autophagy genes have been shown to be tumor prone (7–9). Studies have indicated, however, that the role of autophagy in cancer can depend on tumor type, the stage of tumor development, and the specific genetic mutations associated with disease progression (10–13). The current consensus is that autophagy has a tumor suppressive role before or in the early stages of tumor development when protecting cellular fidelity is fundamental in preventing cancer initiation and early progression (6, 14). In established tumors, however, autophagy generally appears to have a tumor supportive role (6, 14). Within the highly stressed environment of an established tumor, it is considered that autophagy mitigates this stress to permit tumor cell survival under these conditions.

A tumor type in which autophagy has been studied extensively is pancreatic ductal adenocarcinoma (PDAC). Using a genetically engineered mouse model (GEMM) of PDAC, it was reported that PDAC development driven by mutant KRAS was dependent on autophagy (10, 15, 16). This was, however, found to be different in PDAC driven by KRAS and the absence of p53 in which the loss of autophagy was tumor-promoting (10). In contrast to these findings, studies of patient-derived xenografts (PDX) formed in immuno-compromised mice indicated that inhibition of autophagy impaired tumor formation irrespective of p53 status (16). In human PDAC when p53 is perturbed, the *TP53* gene is usually mutated and retained rather than deleted/silenced, and the genetic lesions associated with the disease occur at focal sites within the adult pancreas rather than being expressed from embryonic development as is the case with most GEMM models (17). We therefore decided to analyze autophagy—by conditional deletion of the essential autophagy gene *Atg7*—in a mouse model of PDAC involving alleles for mutant *Kras^G12D^* and mutant *Trp53^172H^*, which are recombined in the adult using an inducible *Cre* recombinase driven by the pancreas selective promoter *Pdx1*. These mice undergo the full spectrum of tumor development from initiation to metastatic disease and our studies using these animals provide further insights into the relationship between *Trp53* and loss of *Atg7* and also highlight disparate roles for ATG7 and autophagy during tumor development.

## Results

### Homozygous Deletion of *Atg7* in the Adult Pancreas Reduces Overall Survival and Causes Tissue Dysfunction.

To examine the role of autophagy in the development of pancreatic cancer in a model that best mirrors the human disease, we crossed conditional *Atg7* knockout mice (*Atg7^fl^*^/^*^fl^*) to mice expressing mutant *Kras* (*Kras^G12D^*^/+^) and mutant *Trp53* (*Trp53^R172H^*^/+^)—two mutational events frequently associated with PDAC development (17). Recombination of these alleles was achieved by an inducible *Pdx1-Cre^ER^* recombinase for pancreas-specific knockout or expression in adult animals, as would occur during the normal etiology of PDAC. We generated three cohorts of mice with differing *Atg7* status, *Atg7*^+/+^, *Atg7*^+/−^, and *Atg^7^*^−/−^, in combination with *Kras^G12D^*^/+^ and *Trp53^R172H^*^/+^ expression in the pancreas (*SI Appendix*, Fig. S1*A*). In addition, as mutant *Kras* is required for tumor formation in this model, we generated similar cohorts, but lacking mutant *Kras* (*Pdx1-Cre^ER^ Trp53^R172H^*^/+^ *Atg7*^+/+^, *Atg7*^+/−^, or *Atg7*^−/−^) to control for any phenotypes that were not associated with PDAC development (*SI Appendix*, Fig. S1*A*).

**Fig. 1.**
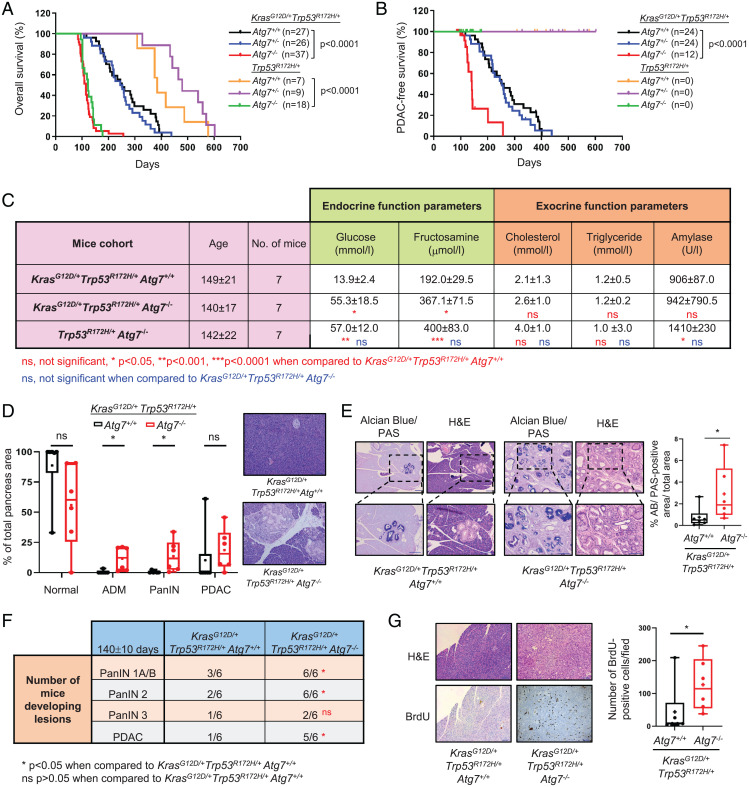
Homozygous deletion of ATG7 in the pancreas reduces overall survival and causes tissue dysfunction. (*A*) Kaplan-Meier plot comparing overall survival of *Atg7*^+/+^ (black), *Atg7*^+/−^ (blue) and *Atg7*^−/−^ (red) *Kras^G12D^*^/+^, *Trp53^R172H^*^/+^ cohorts, and *Atg7*^+/+^ (orange), *Atg7*^+/−^ (purple) and *Atg7*^−/−^ (green), *Trp53^R172H^*^/+^ cohorts upon *Pdx1-Cre^ER^*-mediated recombination with tamoxifen treatment. (*B*) Kaplan-Meier plot comparing PDAC-free survival of the same mice cohorts as (*A*). (*C*) Biochemical analysis of pancreatic function of the indicated genotypes. Blood plasma levels of glucose and fructosamine were measured to assess endocrine function. Blood plasma levels of cholesterol, triglycerides and amylase were measured to assess exocrine function. Values are expressed as median ± interquartile range (Q3–Q1). Kruskal-Wallis test was used for statistics. Stats comparing *Kras^G12D^*^/+^ *Trp53^R172H^*^/+^ *Atg7*^−/−^ or *Trp53^R172H^*^/+^ *Atg7*^−/−^ with *Kras^G12D^*^/+^ *Trp53^R172H^*^/+^ *Atg7*^+/+^ are indicated in red, while stats comparing *Trp53^R172H^*^/+^ *Atg7*^−/−^ with *Kras^G12D^*^/+^ *Trp53^R172H^*^/+^ *Atg7*^−/−^are indicated in blue. ns, not significant *P < 0.05, **P < 0.01, ***P < 0.001. (*D*) Distribution of normal pancreas, precursor lesions (ADM and PanIN) and pancreatic ductal adenocarcinoma (PDAC) within the pancreata of *Atg7*^+/+^ (*n* = 6) and *Atg7*^−/−^ (*n* = 6) *Kras^G12D^*^/+^, *Trp53^R172H^*^/+^ mice at 140 ± 10 d. Data are presented as percentage of area of each tissue type over total pancreata area. Mann-Whitney test was used for statistics. ns, not significant; *P < 0.05. Representative images of pancreas sections stained with H&E that was used to quantify the different lesions. (Scale bars: 100 μm.) (*E*) Representative images of serial sections of pancreata from *Atg7*^+/+^ and *Atg7*^−/−^ *Kras^G12D^*^/+^, *Trp53^R172H^*^/+^ mice at 140 ± 10 d stained with Alcian blue/PAS and H&E. (Scale bars: 100 μm.) Box plot shows the quantification of Alcian blue/PAS-stained PanINs, presented as percentage over total pancreata area (*n* = 6 mice per genotype). Mann-Whitney test was used for statistics. *P < 0.05. (*F*) Table indicating the number of mice developing PanIN 1A/1B, PanIN 2, PanIN 3, or PDAC in the *Atg7*^+/+^ and *Atg7*^−/−^ *Kras^G12D^*^/+^, *Trp53^R172H^*^/+^ cohorts (140 ± 10 d). Data are presented as number of mice developing the respective lesions over total number of mice in each cohort. χ^2^ test was used for statistics, *P < 0.05; ns, not significant. (*G*) Representative images of serial sections of pancreata from *Atg7*^+/+^ and *Atg7*^−/−^ *Kras^G12D^*^/+^, *Trp53^R172H^*^/+^ mice at 140 ± 10 d stained with H&E or BrdU. (Scale bars: 100 μm.) Box plot shows quantification of BrdU-stained nuclei per field of view (*n* = 6 mice for each genotype). Mann-Whitney test was used for statistics. *P < 0.05.

Similar to previous studies using constitutive *Pdx1-Cre* (10), a mosaic deletion of *Atg7* was achieved with the *Pdx1-Cre^ER^* recombinase when *Kras^G12D^*^/+^ *Trp53^R172H^*^/+^ mice homozygous for a floxed allele of *Atg7* were induced with tamoxifen treatment at 7–8 wk of age (*SI Appendix*, Fig. S1*B*). This was accompanied by a strong accumulation of cytoplasmic nonpunctate LC3 and p62 (*Bottom panels* in *SI Appendix*, Fig. S1*B* and *C*), indicating a lack of autophagy in the recombined subpopulation of cells (18). Upon aging of the *Kras^G12D^*^/+^ *Trp53^R172H^*^/+^ *Atg7*^+/+^, *Kras^G12D^*^/+^ *Trp53^R172H^*^/+^ *Atg7*^+/−^, and *Kras^G12D^*^/+^ *Trp53^R172H^*^/+^ *Atg7*^−/−^cohorts, we observed that the overall survival of *Atg7*^−/−^ animals was significantly worse than that of the *Atg7*^+/+^ or *Atg7*^+/−^ cohorts (*P* < 0.0001; Fig. 1*A*), with an overall median survival of 114 d for *Atg7*^−/−^ animals compared with 251 and 249 d for the *Atg7*^+/+^ or *Atg7*^+/−^cohorts, respectively (*SI Appendix*, Table S1). Upon necropsy of the mice, we observed the majority of the *Atg7*^+/+^ or *Atg7*^+/−^ mice (24/27 for *Atg7*^+/+^ and 24/26 for *Atg7*^+/−^) exhibited fully developed PDAC, which was only present in a third of the *Atg7*^−/−^cohort (12/37 mice) (Fig. 1*B* and *SI Appendix*, Table S2).

We were therefore interested to identify the cause of early death in the *Kras^G12D^*^/+^ *Trp53^R172H^*^/+^ *Atg7*^−/−^ animals without evidence of PDAC (25 of 37 mice), and we noted an increased amount of wet bedding in these cages, which we considered was due to increased micturition—a typical sign of diabetes. In addition, early death and excessive urination were also observed in our *Trp53^R172H^*^/+^ *Atg7*^−/−^ cohort (Fig. 1*A* and *SI Appendix*, Table S1). Importantly, this cohort lacks mutant *Kras* and therefore does not develop PDAC (Fig. 1*B* and *SI Appendix*, Table S2). Moreover, the accelerated death observed in the *Trp53^R172H^*^/+^ *Atg7*^−/−^ mice was similar to that observed in the *Kras^G12D^*^/+^ *Trp53^R172H^*^/+^ *Atg7*^−/−^ cohort (*P* < 0.0001) (green and red curves in Fig. 1*A*). Taken together, these data indicate that excessive urination and early death observed upon deletion of *Atg7* were not associated with PDAC development. Previously, we found that embryonic deletion of *Atg7* or *Atg5* (another essential autophagy gene) using the constitutive *Pdx1-Cre* recombinase gave rise to a diabetic phenotype in a large proportion of offspring (10). Pancreatic β cell-specific deletion of *Atg7* has also been reported to cause the degeneration of islets and impairment of glucose tolerance (19). We considered that the use of an inducible *Cre* recombinase, which is activated in adult tissue, might limit this pathology. However, to test if diabetes was still occurring in inducible *Cre* cohorts, we analyzed plasma samples from age-matched *Kras^G12D^*^/+^ *Trp53^R172H^*^/+^ *Atg7*^+/+^ and *Kras^G12D^*^/+^ *Trp53^R172H^*^/+^ *Atg7*^−/−^ mice for pancreatic endocrine and exocrine parameters (Fig. 1*C*). These biochemical analyses revealed an impairment in endocrine function (elevated glucose and fructosamine levels), but not exocrine function (no alterations in cholesterol, triglycerides, and amylase levels). Similar results were also observed in *Trp53^R172H^*^/+^ *Atg7*^−/−^ mice that lack mutant *Kras* (Fig. 1*C*), indicating that the impairment of endocrine function was likely related to loss of autophagy rather than PDAC development. In addition, islet tissue destruction was evident in hematoxylin and eosin (H&E)-stained *Kras^G12D^*^/+^ *Trp53^R172H^*^/+^ *Atg7*^−/−^ and *Trp53^R172H^*^/+^ *Atg7*^−/−^ pancreata (*SI Appendix*, Fig. S2). These data therefore show the importance of ATG7 in preserving endocrine function in the adult pancreas and that the deletion of *Atg7* in adult tissue also results in a pancreatic insufficiency phenotype, similar to what is observed when *Atg7* or *Atg5* are deleted in the embryonic stage (10).

### Homozygous and Hemizygous Deletion of *Atg7* in the Pancreas Accelerates Pancreatic Cancer Initiation.

As a large proportion of the *Kras^G12D^*^/+^ *Trp53^R172H^*^/+^ *Atg7*^−/−^ cohort died early due to pancreatic insufficiency, this limited our ability to accurately analyze the impact of losing autophagy on pancreatic cancer development in this model. As a result, we decided to sacrifice *Kras^G12D^*^/+^ *Trp53^R172H^*^/+^ *Atg7*^+/+^ and *Kras^G12D^*^/+^ *Trp53^R172H^*^/+^ *Atg7*^−/−^ mice at a specific time-point (140 ± 10 d) to enable a better comparison of the disease development between both cohorts. This analysis revealed that at 140 d, *Atg7*^−/−^ mice have a higher incidence of acinar-to-ductal metaplasia (ADM) and pancreatic intraepithelial neoplasia (PanIN) when compared with *Atg7*^+/+^ mice despite having no significant difference in pancreas weight (Fig. 1*D* and *SI Appendix*, Fig. S3). An increase in precancerous PanIN lesions was also evident from the observed higher levels of mucin stain Alcian blue/periodic acid-Schiff (PAS) in the pancreas of *Atg7*^−/−^ mice (Fig. 1*E*). Notably, there were significantly higher numbers of mice developing PanIN and PDAC lesions in the *Atg7*^−/−^ cohort when compared with the *Atg7*^+/+^ cohort (Fig. 1*F*). In an attempt to explain these observations, we assessed the impact of losing *Atg7* on pancreatic cell proliferation by 5-bromo-2′-deoxyuridine (BrdU) labeling and immunohistochemistry (IHC) staining and found *Atg7*^−/−^ tissues to display a significantly higher number of BrdU-positive cells when compared with *Atg7*^+/+^ tissues (Fig. 1*G*), indicating that pancreatic tissue becomes hyper-proliferative in the absence of autophagy.

**Fig. 2.**
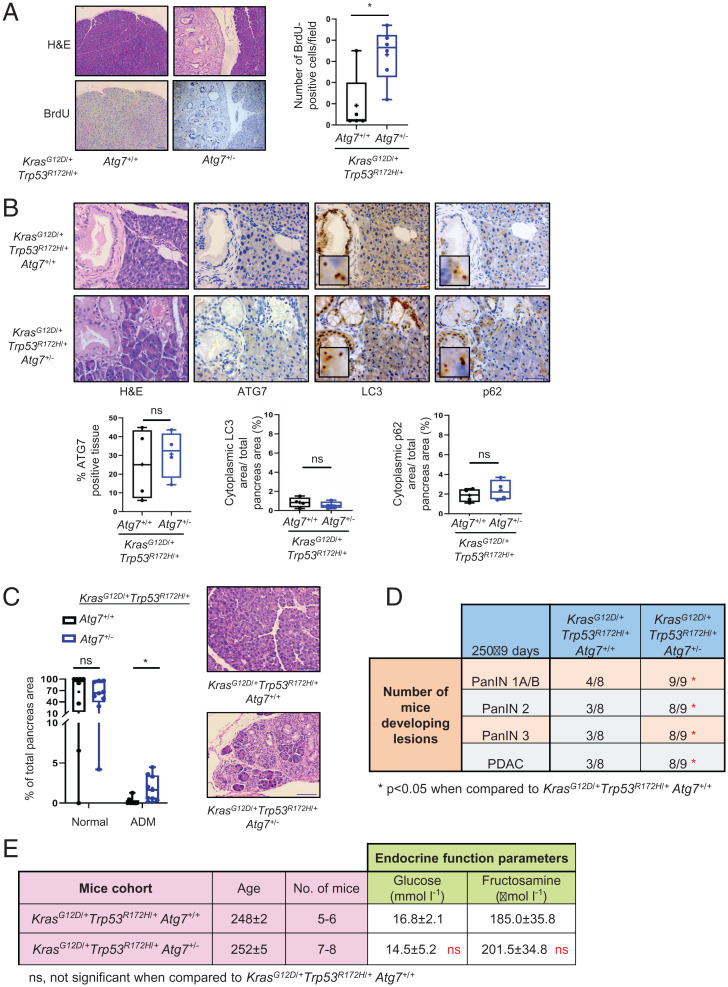
The effect of hemizygous deletion of *Atg7* on pancreatic cancer initiation. (*A*) Representative images of serial sections of pancreata from *Kras^G12D^*^/+^ *Trp53^R172H^*^/+^ *Atg7*^+/+^ and *Kras^G12D^*^/+^ *Trp53^R172H^*^/+^ *Atg7*^+/−^mice at 250 ± 9 d stained with H&E or BrdU. (Scale bars: 100 μm.) Box plot shows quantification of BrdU-stained nuclei per field of view (*n* = 5 mice for *Atg7*^+/+^, *n* = 6 mice for *Atg7*^+/−^). Mann-Whitney test was used for statistics. *P < 0.05. (*B*) Representative images of serial sections of pancreata from *Kras^G12D^*^/+^ *Trp53^R172H^*^/+^ *Atg7*^+/+^ and *Kras^G12D^*^/+^ *Trp53^R172H^*^/+^ *Atg7*^+/−^ mice at 250 ± 9 d stained for H&E, ATG7, LC3, and p62. *Insets* are magnified crops of the images at 7.5× magnification. (Scale bars: 50 μm.) Box plots show the quantification of ATG7, diffused cytoplasmic LC3, and diffused cytoplasmic p62 stainings within the pancreata of *Atg7*^+/+^ (*n* = 4) and *Atg7*^+/−^ (*n* = 4) mice at 250 ± 9 d. Mann-Whitney test was used for statistics. ns, not significant. (*C*) Distribution of normal pancreas and ADM precursor lesions within the pancreata of *Kras^G12D^*^/+^ *Trp53^R172H^*^/+^ *Atg7*^+/+^ (*n* = 8) and *Kras^G12D^*^/+^ *Trp53^R172H^*^/+^ *Atg7*^+/−^ (*n* = 9) mice at 250 ± 9 d. Data are presented as percentage of each region over total pancreata area. Mann-Whitney test was used for statistics. *P < 0.05. Representative images of pancreas sections stained with H&E that were used to quantify the different lesions. (Scale bars: 100 μm.) (*D*) Table indicating the number of mice developing PanIN 1A/1B, PanIN 2, PanIN 3, or PDAC in the *Kras^G12D^*^/+^ *Trp53^R172H^*^/+^ *Atg7*^+/+^ and *Kras^G12D^*^/+^ *Trp53^R172H^*^/+^ *Atg7*^+/−^ cohorts (250 ± 9 d). Data are presented as number of mice developing the respective lesions over total number of mice in each cohort. χ^2^ test was used for statistics, *P < 0.05. (*E*) Biochemical analysis of pancreatic endocrine function (blood plasma levels of glucose and fructosamine) of the indicated genotypes. Values are expressed as median ± interquartile range (Q3–Q1). Mann-Whitney test was used for statistics. ns, not significant when compared with *Kras^G12D^*^/+^ *Trp53^R172H^*^/+^ *Atg7*^+/+^.

During our analysis of cell proliferation, we also observed that *Kras^G12D^*^/+^ *Trp53^R172H^*^/+^ *Atg7*^+/−^ mice had higher levels of BrdU staining in their pancreata when compared with *Kras^G12D^*^/+^ *Trp53^R172H^*^/+^ *Atg7*^+/+^ animals without any significant increase in pancreas weight (Fig. 2*A* and *SI Appendix*, Fig. S4). Although these results were similar to what we had observed upon comparison of *Kras^G12D^*^/+^ *Trp53^R172H^*^/+^ *Atg7*^+/+^ and *Kras^G12D^*^/+^ *Trp53^R172H^*^/+^ *Atg7*^−/−^ mice (Fig. 1*G*), it was surprising because previous studies have shown that hemizygosity of Atg7 does not lead to loss of autophagy (4). To test if this was the case in our model, we stained pancreatic tissue from *Atg7*^+/+^ and *Atg7*^+/−^ mice for ATG7, LC3, and p62. This revealed, different to what was observed from our analysis of *Atg7*^−/−^ mice (*SI Appendix*, Fig. S1*B* and *C*), that loss of one allele of *Atg7* in *Atg7*^+/−^ mice was not accompanied by differences in LC3 or p62 (Fig. 2*B*), suggesting that autophagy was active in the pancreata of this cohort. Upon closer analysis, we also observed that hemizygous loss of *Atg7* appeared to cause an increase in the incidence of ADM metaplasia—the process thought to precede PanIN formation (Fig. 2*C*). As these observations might predict an increase in precancerous lesions and perhaps also PDAC development, we performed a more detailed analysis of pancreatic tissue harvested from *Atg7*^+/+^ and *Atg7*^+/−^ mice at 250 ± 9 d. Notably, we also found a significantly higher number of mice developing PanIN and PDAC lesions in the *Atg7*^+/−^ cohort when compared with the *Atg7*^+/+^ cohort (Fig. 2*D*). This confirmed that loss of one allele of *Atg7* in the pancreata of *Atg7*^+/−^ mice not only caused an increase in incidence of PanIN lesions, but also PDAC (Fig. 2*D*). Interestingly however, this did not impact overall survival or PDAC-free survival in this model (Fig. 1 *A* and *B*, and *SI Appendix*, Tables 1 and 2); suggesting that the biggest contributing factor to death due to loss of *Atg7* in this model is not PDAC, but diabetes and pancreatic insufficiency. Indeed, when we analyzed plasma samples from age-matched *Atg7*^+/+^ and *Atg7*^+/−^ mice for pancreatic endocrine parameters, the results revealed no marked changes in endocrine (elevated glucose and fructosamine levels) function (Fig. 2*E*) compared with what was previously observed upon complete deletion of *Atg7* (Fig. 1*C*). When taken together, these data therefore suggest that reduced levels of ATG7 can affect precancerous lesions and PDAC formation by a mechanism independent of its role in the regulation of autophagy.

### Deletion of *Atg7* Reduces the Incidence of PDAC Metastases.

**Fig. 3.**
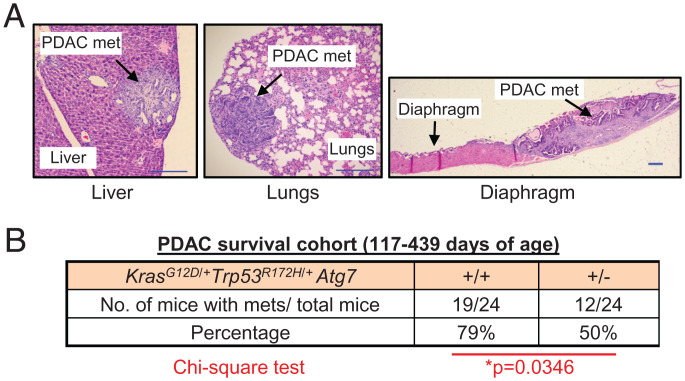
Hemizygous deletion of *Atg7* in the pancreas reduces metastatic potential of PDAC driven by mutant *Kras* and mutant *Trp53*. (*A*) Representative images of the types of PDAC metastases (liver, lungs, and diaphragm) observed in the cohorts of this study. (Scale bars: 200 μm.) (*B*) Number of mice with PDAC metastases (liver, lungs, or diaphragm) in the *Kras^G12D^*^/+^ *Trp53^R172H^*^/+^ *Atg7*^+/+^ and *Kras^G12D^*^/+^ *Trp53^R172H^*^/+^ *Atg7*^+/−^ cohorts. χ^2^ test was used for statistics.

An additional advantage of this mouse model of PDAC driven by *Kras^G12D^*^/+^ *Trp53^R172H^*^/+^ is that it undergoes metastasis to other organs including liver, lungs, and diaphragm (Fig. 3*A*). At diagnosis, the majority of patients present with metastatic disease and this contributes to treatment failure and increased mortality (20). Because our *Kras^G12D^*^/+^ *Trp53^R172H^*^/+^ *Atg7*^+/−^ mice exhibit enhanced PDAC development, but do not die due to pancreatic insufficiency, we considered that we could evaluate the effects of *Atg7* hemizygosity on the development of PDAC metastasis. As we had observed that hemizygous deletion of *Atg7* appeared to enhance PDAC progression, we logically considered this may also lead to increased metastasis. However, our analysis revealed that deletion of one allele of *Atg7* actually caused a significant decrease in metastatic potential in this model (Fig. 3*B*). Of note, however, while the overall incidence of metastatic disease is reduced in *Atg7* hemizygous animals, those that do undergo metastasis present PDAC lesions in the same secondary organs—liver, lungs, and diaphragm—as those wild-type for *Atg7*.

### Hemizygous Deletion of *Atg7* Alters Metabolism and Impairs Cell Invasion.

**Fig. 4.**
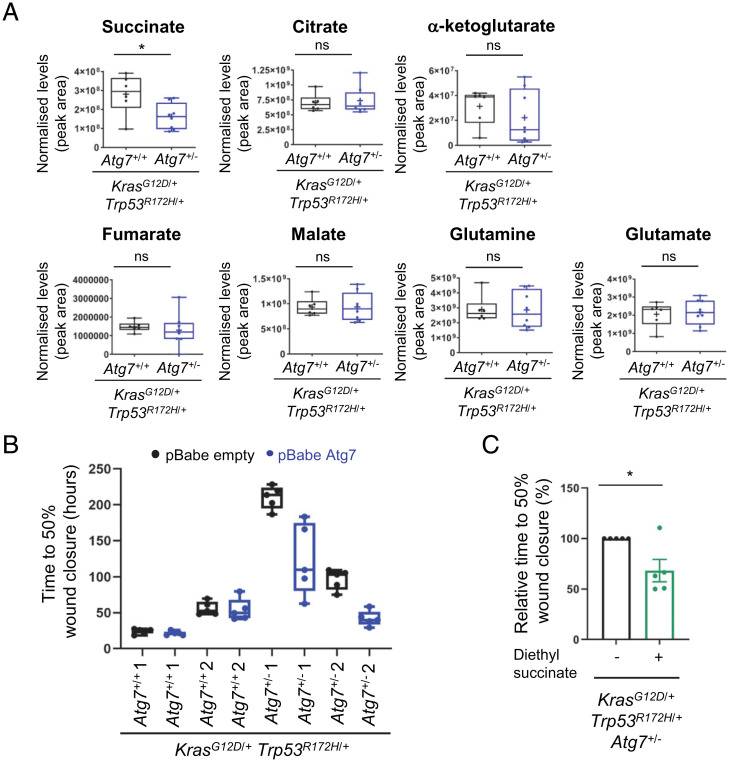
PDAC tumors with hemizygous deletion of *Atg7* have reduced succinate levels and *Atg7* re-expression or diethyl succinate treatment restores invasive potential of *Kras^G12D^*^/+^, *Trp53^R172H^*^/+^ *Atg7*^+/−^ PDAC cells. (*A*) Box plots showing levels of succinate and other TCA cycle intermediates in PDAC tumor tissues from *Kras^G12D^*^/+^ *Trp53^R172H^*^/+^ *Atg7*^+/+^ (*n* = 6) and *Kras^G12D^*^/+^ *Trp53^R172H^*^/+^ *Atg7*^+/−^ (*n* = 8) animals measured by LC-MS. Mann-Whitney test was used for statistics. *P < 0.05; ns, not significant. (*B*) Box plots showing that *Kras^G12D^*^/+^ *Trp53^R172H^*^/+^ *Atg7*^+/−^ cells is less invasive compared with their *Kras^G12D^*^/+^ *Trp53^R172H^*^/+^ *Atg7*^+/+^ counterparts but ATG7 re-expression restores invasive potential of *Kras^G12D^*^/+^ *Trp53^R172H^*^/+^ *Atg7*^+/−^ cells as examined by scratch-wound invasion assays. Two cell lines isolated from separate animals were examined for each genotype. These data are representative of three independent experiments (additional data in *SI Appendix*, Fig. S7 *A* and *B*). (*C*) Diethyl succinate treatment improves the ability of *Kras^G12D^*^/+^ *Trp53^R172H^*^/+^ *Atg7*^+/−^ cells to invade as examined by scratch-wound invasion assays. Data are presented as percentage of time taken for 50% wound closure when compared with untreated cells (which are normalized to 100%). Data are from five independent experiments and each data point is a mean of five technical replicates. Error bars are SEM. Paired *t* test was used for statistical analysis. *P < 0.05.

To try and understand the mechanism behind the effects we have observed, we decided to examine the levels of metabolites in *Kras^G12D^*^/+^ *Trp53^R172H^*^/+^ *Atg7*^+/+^ and *Kras^G12D^*^/+^ *Trp53^R172H^*^/+^ *Atg7*^+/−^ tumors. In our previous work (10), we had found several metabolic changes upon biallelic deletion of *Atg7* in a mouse model of pancreas cancer and we reasoned that some of these changes may also occur in *Atg7*^+/−^ tumors. We analyzed metabolites in the glycolysis, tricarboxylic acid (TCA) cycle, and pentose phosphate pathway. This revealed that while there was no change in the majority of metabolites, the levels of succinate were significantly lower in extracts from *Kras^G12D^*^/+^ *Trp53^R172H^*^/+^ *Atg7*^+/−^ tumors when compared with extracts from *Kras^G12D^*^/+^ *Trp53^R172H^*^/+^ *Atg7*^+/+^ tumors (Fig. 4*C* and *SI Appendix*, Fig. S6 *A* and *B*).

As previous studies had reported that succinate can affect metastasis (21), we decided to examine this further. To do this, we generated cell lines from *Kras^G12D^*^/+^ *Trp53^R172H^*^/+^ *Atg7*^+/+^ and *Kras^G12D^*^/+^ *Trp53^R172H^*^/+^ *Atg7*^+/−^ tumors and examined the invasion capacity of these cells in Matrigel-overlaid scratch-wound assays. This revealed, in line with the decreased metastatic potential we had observed in *Kras^G12D^*^/+^ *Trp53^R172H^*^/+^ *Atg7*^+/−^ tumors, that cells hemizygous for *Atg7* had diminished invasive capacity when compared with those wild-type for *Atg7* (Fig. 4*B* and *SI Appendix*, Fig. S7 *A* and *B*). To examine if this effect was definitely related to *Atg7* status, we transduced *Atg7*^+/+^ and *Atg7*^+/−^ cells with a retrovirus expressing *Atg7*. This served to restore the invasive capacity of *Atg7*^+/−^ cells (Fig. 4*B* and *SI Appendix*, Fig. S7 *A* and *B*), but importantly this did not affect the levels of LC3-II or p62 (*SI Appendix*, Fig. S5 *A* and *B*). This therefore shows that ATG7 is not limiting for efficient autophagy in *Atg7*^+/−^ cells and, concordant with our conclusions relating to metastatic potential in vivo, that hemizygosity of *Atg7* affects cellular invasion in an autophagy-independent manner.

Finally, as we had observed that extracts from *Kras^G12D^*^/+^ *Trp53^R172H^*^/+^ *Atg7*^+/−^ tumors had lower levels of succinate when compared with extracts from *Kras^G12D^*^/+^ *Trp53^R172H^*^/+^ *Atg7*^+/+^ tumors, we wanted to know if addition of succinate could affect the invasive capacity of cells derived from *Atg7*^+/−^ tumors. We found that treatment of *Atg7*^+/−^ cells with diethyl succinate (a cell and mitochondrial permeable analog of succinate) could significantly enhance the invasive capacity of *Atg7*^+/−^ cells (Fig. 4*C*), underscoring the connection between this metabolite, the invasive potential, and the allelic status of *Atg7*.

In summary, our findings uncover autophagy-independent roles of ATG7 in both the repression of primary tumor development and in the promotion of metastasis and cellular invasion. These findings also provide insight into studies that utilize deletion of *Atg7* to understand autophagy and highlight additional considerations for the targeting of ATG7 and the reactions it coordinates for the treatment of cancer and equally other forms of disease.

## Discussion

Within this study, we examine the role of autophagy in a model of pancreatic ductal adenocarcinoma, which we believe, out of currently available models, best recapitulates the etiology of the human disease. The model involves alleles of mutant *Kras* and mutant *Trp53*—two key mutational events in human PDAC (17)—which are activated in adult tissue by an inducible *Cre* recombinase. This then results in the formation of pancreatic intraepithelial neoplasia that progress to invasive and metastatic disease within the lifespan of the model, enabling us to examine the impact of the loss of autophagy at all stages of PDAC progression. While it was important to see that the effects of loss of *Atg7* on primary tumor development in this model were similar to our studies on PDAC in mice driven by mutant *Kras* and deletion of *Trp53* (10), it was striking and unexpected to see that hemizygous loss of *Atg7* also had an effect on the number of animals with PanINs and PDAC when compared with animals wild-type for *Atg7*. Intriguingly, we show, in line with previous studies, that *Atg7* hemizygosity does not ablate autophagy (4), as evidenced by the presence of LC3 puncta and levels of p62 comparable to animals wild-type for *Atg7*. This suggests that there are functions of ATG7 in cancer that are autophagy-independent, and which are dependent on the dosage of the gene.

Mechanistically, we found that *Kras^G12D^*^/+^ *Trp53^R172H^*^/+^ *Atg7*^+/−^ tumors had lower levels of succinate when compared with *Kras^G12D^*^/+^ *Trp53^R172H^*^/+^ *Atg7*^+/+^ tumors, and that examination of cell lines derived from *Kras^G12D^*^/+^ *Trp53^R172H^*^/+^ *Atg7*^+/+^ and *Kras^G12D^*^/+^ *Trp53^R172H^*^/+^ *Atg7*^+/−^ tumors showed that hemizygous deletion of Atg7 reduces invasive potential. We were also able to show that “rescue” of *Atg7* hemizygosity in this context by ectopic expression of endogenous *Atg7* can restore this diminished invasive potential while having no effect of the autophagy activity of the cells. This once again affirms that the effects we report here resulting from deletion of one allele of *Atg7* are autophagy-independent. Moreover, we were finally able to show that treatment of cells with diethyl succinate could also significantly enhance the metastatic capacity of *Atg7*^+/−^ cells indicating that there is a link between *Atg7* gene dosage, the levels of this metabolite, invasion and by extrapolation, metastatic potential. While these data serve to provide a mechanistic basis for our observations, some clear questions still remain. First, how does loss of one allele of *Atg7* affect succinate levels and how is invasive capacity affected at the molecular level. Clearly, both of these questions are worthy of greater investigation in future studies.

It is not without precedent that ATG7 has roles that are autophagy-independent. The process of LC3-associated phagocytosis (LAP) (22, 23) and LC3-associated endocytosis (LANDO) (24) require the LC3-conjugation machinery for vesicle formation, and ATG7 has a fundamental role in this process. However, studies have shown that hemizygous loss of *Atg7* does not affect the levels of LC3 conjugation to phosphatidylethanolamine and so it seems unlikely that loss of one allele of *Atg7* will affect LAP or LANDO (4). ATG7 has, however, also been shown to have functions independent of its E3 ligase activity, which is critical for its role in LC3 conjugation. Notable is a study by Lee and colleagues (25), which showed that ATG7 could influence the transcriptional activity of p53 in response to nutrient stress, with activation of p21 being dependent on an interaction between ATG7 and p53 in an autophagy-independent manner. At first, this mechanism might seem a plausible explanation for our results due to the change in proliferative capacity we observe upon loss of *Atg7*, but the activity reported in the study by Lee and co-workers was related to wild-type p53 and our mouse models involve mutant p53. Interestingly, a different study has recently reported the presence of a short form of ATG7 in various tissues (26). This short form lacks the amino acids from the C terminus and as a result cannot function in LC3 conjugation (26). The generation of mice that express this short form of ATG7, but not full-length, would be very interesting and could be utilized to see if this short form retains the tumor suppressive function of ATG7 that is independent of LC3 conjugation as we report here. Despite these interesting reports, the autophagy-independent activity provided by ATG7 that contributes to this haploinsufficient effects we observe are currently unclear, and future studies to explore this function would be undoubtedly worthwhile.

Another important remaining question from this work is whether hemizygosity (or nonfunctional heterozygosity) of *Atg7* and the phenotypes associated with this genotype are relevant to human cancer. To the best of our knowledge, there is no evidence to indicate that one allele of *Atg7* is lost or silenced during tumor development. However, a recent study has shown that *Atg7* can be epigenetically silenced during pancreatitis by a mechanism involving the FOXF1 adjacent noncoding developmental regulatory RNA (FENDRR) and the epigenetic repressor PRC2 (27). It would therefore be interesting to know if this can occur in a way that can cause a reduction in *Atg7* expression that is consistent with a reduction that might occur upon loss or mutation of one allele of *Atg7*. In another study by Nuta and colleagues (28), the authors reported a cancer-associated mutation in LC3 which affects binding to ATG7 and its function in LC3 conjugation. The presence of this mutation would therefore suggest that the autophagy-dependent (and LAP- and LANDO-dependent) and independent functions of ATG7 are separable and further studies of this mutation, especially in comparison with loss of *Atg7*, should yield insights into the different functions of ATG7 in cancer.

In summary, our study shows that hemizygosity of *Atg7* enhances the early stages of PDAC development, but reduces the metastatic potential of PDAC in this model, indicating that there is antagonistic pleiotropy regarding ATG7 functions in PDAC development that are autophagy independent. In addition, in terms of cancer therapy in advanced PDAC, this raises the possibility that lower doses of ATG7-specific inhibitors, that reduce ATG7 function, but do not completely ablate autophagy, may result in therapeutics that could repress metastasis without affecting the beneficial effects of autophagy in normal tissue.

## Materials and Methods

### Animal Experiments.

All animals were housed in a pathogen-free environment with a 12-h light/dark cycle with access to food and water ad libitum. The *Pdx1-Cre^ER^* (MGI:5008261), *Kras^G12D^*^/+^ (MGI:5440073), *Trp53^R172H^*^/+^ (MGI:3039263), and *Atg7^fl^*^/^*^fl^* (MGI:3587769) mice strains have been previously described (4, 29–31). *Pdx1-Cre^ER^*, *Kras^G12D^*^/+^, *p53^R172H^*^/+^, *Atg7^fl^*^/^*^fl^*, and *Pdx1-Cre^ER^*, *p53^R172H^*^/+^, *Atg7^fl^*^/^*^fl^* compound mice were maintained on a mixed C57bl/6 background. Experiments were designed and carried out in compliance with UK Home Office regulations (project license P54E3DD25) and approved by the Animal Welfare and Ethical Review Body of The University of Glasgow. Mice were randomized into experimental cohorts with no blinding applied. All experimental mice express *Pdx1-Cre^ER^* and recombination was induced at 7–8 wk using a single intraperitoneal injection of 80 mg/kg tamoxifen for 3 consecutive days. Mice were subsequently aged and regularly monitored until clinical signs (distended abdomen, hunching, high gait, and/or weight loss) developed, or killed at specified time points (as indicated). Genotyping was performed by Transnetyx, Inc.

### Cell Culture.

Tumor cell lines were isolated from mice PDAC tissue and cultured as previously described (10). Cells were maintained in Dulbecco’s modified Eagle’s medium (DMEM) supplemented with 10% fetal bovine serum (FBS), 2 mM glutamine, 100 mg/mL streptomycin, and 100 U/mL penicillin (referred as complete media) and cultured in humidified atmosphere at 5% CO_2_. PDAC cells expressing mAtg7 were generated by infecting with virus containing pBabe-blast-empty or pBabe-blast-mAtg7 and selecting with 5 μg/mL blasticidin for 7 d. pBabe-blast-mAtg7 was generated by PCR from the I.M.A.G.E. clone for murine *Atg7* (kind gift from Simon Wilkinson, Institute of Genetics and Cancer, University of Edinburgh, UK) subsequently digested with BamHI and EcoRI, and cloned in to the BamHI and EcoRI sites of pBabe-blast.

### Blood Biochemistry.

For analyses of pancreatic function, mice were humanely euthanized and blood was harvested by cardiac puncture into heparinized tubes. Centrifugation was next performed at 1,500 × *g* for 5 min and plasma was separated and stored at −80 °C until required. Analysis of plasma levels of glucose, fructosamine, cholesterol, triglycerides, and amylase were carried out by the Veterinary Diagnostic Services Laboratory, University of Glasgow, UK.

### BrdU Quantification.

For assessment of cell proliferation by BrdU labeling, mice were administered a single intraperitoneal injection of 0.75 mg BrdU (Sigma; B5002) in 250 μL PBS 2 h prior to necropsy. IHC staining for BrdU was performed as mentioned below. For quantification, BrdU-positive cells were counted manually from an average of at least five random fields of view per tissue. The number of mice counted is specified in the respective figure legends.

### Immunohistochemistry.

Whole pancreatic, liver, lung, and diaphragm tissues were fixed in 10% neutral-buffered formalin (Solmedia; FORM011) immediately after harvest for at least 24 h prior to paraffin embedding. Alcian Blue/PAS, H&E, and IHC staining was performed on 4-µm formalin-fixed paraffin-embedded (FFPE) sections which had previously been baked in the oven at 60 °C for 2 h.

FFPE sections underwent a standard H&E protocol performed on a Leica ST5020 autostainer according to manufacturer’s instructions.

The following antibodies were stained on a Dako AutostainerLink48: BrdU (347580, BD Biosciences), LC3 (0231-100; Nanotools), and p62 (BML-PW986, Enzo). Sections for p62 underwent manual dewaxing with xylenes, graded alcohol, and then washed in tap water before undergoing heat-induced epitope retrieval (HIER). HIER was performed on a Dako PT module where the 4-µm sections were heated to 97 °C for 20 min in appropriate retrieval buffer. Sections for BrdU and LC3 were dewaxed and retrieved using Target Retrieval Solution, high pH (Agilent; K8004) and sections for p62 were retrieved using Antigen Unmasking Solution, pH6 (Vector Labs; H-3300). After epitope retrieval, sections were rinsed in Flex wash buffer (Agilent; K8007) prior to being loaded onto the autostainer. All sections underwent peroxidase blocking (Agilent; S2023), rinsed with wash buffer before sections for BrdU and LC3 staining received mouse immunoglobulin (Ig)-blocking solution (Vector Labs; MKB-2213) for 20 min prior to rinsing with wash buffer. Each primary antibody was applied at a previously optimized dilution (BrdU, 1/250; LC3, 1/100; p62, 1/250; Atg7, 1/2,000) for 40 min at room temperature. The sections were then rinsed with wash buffer before application of either mouse or rabbit envision secondary antibody (Agilent; K4001 or K4006) for 30 min at room temperature. Sections were subsequently rinsed with wash buffer before applying Liquid DAB (Agilent; K3468). The sections were then washed in water, counterstained with Haem Z (CellPath; RBA-4201-00A) and mounted using DPX mountant (CellPath; SEA-1304-00A).

IHC staining for Atg7 was performed manually. Sections for Atg7 underwent manual dewaxing with xylenes, graded alcohol, and then washed in tap water before undergoing HIER for 30 min in boiling Tris-EDTA retrieval buffer, pH 9.0 (Abcam; ab93684). Slides were rinsed in Tris-buffered saline (TBS) and blocked for 30 min at room temperature with 5% vol/vol goat serum (Agilent; X0907) and 2.5% wt/vol bovine serum albumin (Fisher Scientific; BP9701-100) in TBS, 0.05% Tween 20. The sections were incubated with Atg7 antibody (Abcepta; AP1813D,) at 1/2,000 dilution at 4 °C overnight. The sections were then rinsed with TBS before peroxidase blocking (Agilent; S2023) for 20 min and subsequent application of rabbit secondary antibody (Agilent; K4006) for 30 min at room temperature. Sections were rinsed with TBS before applying Liquid DAB (Agilent; K3468). The sections were then washed in water, counterstained, and mounted as described above.

Alcian blue/PAS staining was performed manually using a standard Alcian blue/PAS histology protocol. Briefly, dewaxed sections were stained with Alcian blue (Sigma; A3157) for 30 min, treated with periodic acid (Surgipath Leica; 3803812) for 10 min, and finally with Schiff’s reagent (Surgipath Leica; 3803800) for 20 min (water washes were performed between treatments and after the Schiff’s reagent staining). Sections were dehydrated by treating with increasing concentrations of ethanol (70–100%) and xylene and subsequently mounted as described above.

All images were taken using a Zeiss AX10 (light microscope) at a 20× or 40× magnification.

### Quantification of Atg7, LC3, p62, and Alcian Blue/PAS Staining, Distribution of Pancreatic Lesions, and PanIN Number.

Whole pancreas tissue sections were scanned with a Aperio AT2 slide scanner (Leica Biosystems, UK) at 40× magnification.

Atg7, diffused cytoplasmic LC3, and diffused cytoplasmic p62 staining were quantified in normal pancreas and PanIN-containing tissue of the entire pancreas using the area quantification v2.2.4 algorithm of the HALO image analysis platform (Indica Labs). The data are presented as percentage of tissue area stained with Atg7, diffused cytoplasmic LC3, or diffused cytoplasmic p62 over total area of the pancreas section.

Mucin-producing PanINs were quantified by measuring Alcian blue/PAS-stained tissue in the entire pancreas using the area quantification v2.1.7 algorithm of the HALO image analysis platform (Indica Labs). The data are presented as percentage of tissue area showing strong Alcian blue/PAS staining over total area of the pancreas section.

The distribution of normal pancreas, precursor lesions (ADM and PanIN) and PDAC within each pancreas was quantified by manually annotating and measuring the area of each tissue type within the entire pancreas section using the HALO image analysis platform. The data are presented as percentage of the area of normal tissue or precursor/PDAC lesion over total area of the pancreas section.

Identification of the different grades of PanIN and PDAC was performed by visual examination of the lesions in the entire pancreas section using the slide viewing software, Aperio ImageScope (Leica Biosystems). The incidence of each type of lesion in the *Kras^G12D^*^/+^ *Trp53^R172H^*^/+^ *Atg7*^+/+^, *Kras^G12D^*^/+^ *Trp53^R172H^*^/+^ *Atg7*^+/−^, and *Kras^G12D^*^/+^ *Trp53^R172H^*^/+^ *Atg*^−/−^cohorts were recorded.

The number of mice counted for each analysis is specified in the respective figure legends.

### Scratch-Wound Invasion Assay.

96-well ImageLock plates (Essen Bioscience; 4379) were coated with 10% Corning Matrigel growth factor reduced basement membrane matrix (Merck; CLS356231) diluted in complete media overnight at 37  °C. PDAC cells were seeded into the Matrigel-coated plates (35,000 cells in 100 μL complete media per well) and left to adhere overnight at 37  °C. The monolayer of cells was then wounded with the Woundmaker tool (Essen Bioscience; 4563) and washed twice with media to remove unwanted cell debris. Subsequently, 50 μL of 50% Corning Matrigel growth factor reduced basement membrane matrix (CLS356231) diluted in complete media (with or without 5 mM diethyl succinate treatment (Merck; 112402) was overlaid onto the wounded monolayer and left to polymerize for 1 h at 37  °C. After 1 h, 100 μL complete media (with or without drug treatment) was added into each well and plates were imaged every hour for 4 d using the IncuCyte S3 instrument (Essen Bioscience). Results are presented as the time point at which the wound confluence of the samples is 50% (*T*_max_1/2).

### Western Blotting.

Cells were lysed in the buffer containing 50 mM Hepes (Merck; H3375), 150 mM NaCl (Merck; S9888), 10 mM EDTA (Merck; E5134), 100 mM NaF (Merck; 201154), 10 mM Na_4_P_2_O_7_ (Fisher Scientific; S/6140/53), 1% Triton X-100 (Merck; T9284), 0.1% SDS (Fisher Scientific; S/P530/53), and protease inhibitor mixture (Merck; 04693124001) for 30 min on ice prior to centrifugation for 15 min at 20,000 × *g* at 4 °C. Twenty micrograms of protein was separated by SDS-PAGE and transferred to PVDF membranes using standard techniques. Membranes were probed using standard immunoblotting techniques with the following antibodies: LC3B (Cell Signaling Technology; 2775), Actin (Cell Signaling Technology; 4970), Atg7 (Cell Signaling Technology; 8558), p62 (Enzo Life Sciences; BML-PW9860), anti-mouse IgG HRP-linked (Cell Signaling Technology; 7076), or anti-rabbit IgG HRP-linked (Cell Signaling Technology; 7074).

### Liquid Chromatography–Mass Spectrometry Analysis.

#### PDAC tumor tissue extraction.

PDAC tumor tissues harvested from mice were homogenized at 20 mg/mL with the extraction solvent (50% methanol, 30% acetonitrile, 20% Milli-Q water) in ceramic bead-containing tubes (Stretton Scientific Ltd.; CK28) using Precellys 24 homogenizer (Stretton Scientific Ltd.). Homogenization was carried out in 3 × 30-s cycles, with a 30-s gap between each cycle. Homogenized samples were transferred to microcentrifuge tubes and centrifuged at 16,100 × *g* for 10 min at 4 °C. The supernatants were transferred into high-performance liquid chromatography (HPLC) vials and stored at −80 °C before liquid chromatography–mass spectrometry (LC-MS) analysis.

The LC-MS analysis of metabolites in PDAC tissues was carried out as previously described (32). A Q Exactive Orbitrap mass spectrometer (Thermo Scientific) with electrospray (ESI) ionization coupled with a Thermo Ultimate 3,000 HPLC system was used. Metabolite separation was carried out using a ZIC-pHILIC column (SeQuant, 150 × 2.1 mm, 5 µm, Merck KGaA) held at a column temperature of 45 °C. Initial mobile phase consisted of 20% 20-mM ammonium carbonate, pH 9.2, and 80% acetonitrile, and metabolites were separated over a 15-min gradient decreasing the acetonitrile content to 20% at a flow rate of 200 μL/min. All metabolites were detected across a mass range of 75 to 1,000 *m/z* at a resolution of 35,000 (at 200 *m/z*) and using polarity switching to enable both positive and negative ions to be detected in the same run. The mass accuracy was below 5 ppm. Data were acquired with Xcalibur and processed using TraceFinder v4.1 software (Thermo Scientific), where metabolites were identified by the exact mass of the singly charged ion and by known retention time on the LC-MS run.

### Box-and-Whisker Plots and Statistics.

Box-and-whisker plots were used to present some of the data in this study. The box spans the interquartile range, from lower (Q1) to upper (Q3) quartile, while the median is marked by a line in the box. The whiskers indicate the minimum and maximum values of each dataset. If not overlapping with other data points, the mean is also indicated by a “+” within the box.

Statistical analysis of data was performed using the GraphPad Prism software. The statistical tests used to analyze the data are indicated in the corresponding figure legends. Results were considered statistically significant when *P value < 0.05, **P value < 0.01, or ***P value < 0.001 (ns, no significance).

## Supplementary Material

Supplementary File

## Data Availability

All data have been deposited in Enlighten: Research Data (https://doi.org/10.5525/gla.researchdata.1295) (33).
