# Supplementary material for: ATG7 is a haploinsufficient repressor of tumor progression and promoter of metastasis
Source: Proc Natl Acad Sci U S A. 2022 Jul 6;119(28):e2113465119. doi: 10.1073/pnas.2113465119 (PMC9282388; doi:10.1073/pnas.2113465119)
Supplement: Supplementary File [file pnas.2113465119.sapp.pdf]

Supplementary Information Appendix

Long et al. "Atg7 is a haploinsufficient repressor of tumor progression and promoter of metastasis"

Supplemental Figure and Table Legends

Supplemental Figure 1.

Mice cohorts used in the study. (a) Schematic of the experimental mice cohorts and the genetically modified alleles expressed (b) Representative images of serial sections of pancreata from *Kras*$^{G12D/+}$ *Trp53*$^{R172H/+}$ *Atg7*$^{+/+}$ and *Kras*$^{G12D/+}$ *Trp53*$^{R172H/+}$ *Atg7*$^{-/-}$ animals stained for H&E, ATG7, LC3 and p62. Insets are magnified crops of the images at x15 magnification. Scale bars: 100 µm. (c) Box plots show the quantification of ATG7, diffused cytoplasmic LC3 and diffused cytoplasmic p62 stainings within the pancreata of *Atg7*$^{+/+}$ (n=6) and *Atg7*$^{-/-}$ (n=6) mice at 140±10 days. Mann-Whitney test was used for statistics. *p<0.05,**p<0.01.

Supplemental Figure 2.

Representative images of pancreatic endocrine tissue from *Kras*$^{G12D/+}$ *Trp53*$^{R172H/+}$ *Atg7*$^{+/+}$, *Kras*$^{G12D/+}$ *Trp53*$^{R172H/+}$ *Atg7*$^{-/-}$ and *Trp53*$^{R172H/+}$ *Atg7*$^{-/-}$ mice stained with H&E. Morphological destruction is observed in both *Atg7*$^{-/-}$ islets compared to *Atg7*$^{+/+}$. Scale bars: 100 µm.

Supplemental Figure 3.

Whole pancreata from *Atg7*$^{+/+}$ (n=6) or *Atg7*$^{-/-}$ (n=8) *Kras*$^{G12D/+}$*, Trp53*$^{R172H/+}$ mice sacrificed at 140±10 days, were isolated, weighed, and normalized against body weight. Mann-Whitney test was used for statistics. ns, not significant.

Supplemental Figure 4.

Whole pancreata from *Atg7*$^{+/+}$ (n=6) or *Atg7*$^{+/-}$ (n=8) *Kras*$^{G12D/+}$, *Trp53*$^{R172H/+}$ mice sacrificed at 250±9 days, were isolated, weighed, and normalized against body weight. Mann-Whitney test was used for statistics. ns, not significant.

Supplemental Figure 5.

(a-b) Cell lines isolated from PDAC tumours from *Kras*$^{G12D/+}$ *Trp53*$^{R172H/+}$ *Atg7*$^{+/+}$ (*Atg*7$^{+/+}$) and *Kras*$^{G12D/+}$ *Trp53*$^{R172H/+}$ *Atg7*$^{+/-}$ (*Atg*7$^{+/-}$) animals were infected with empty control vector (pBabe) or murine Atg7 (pBabe Atg7), analysed using SDS-PAGE and western blotted for ATG7, p62 and LC3. β-actin was used at a loading control. Western blot shows no marked differences in LC3 II or p62 levels between control (lanes 1-5) or ATG7-overexpressed cell lines (lanes 6-10). The western blot is representative of 3 independent experiments.

Supplemental Figure 6.

Box plots showing levels of glycolytic and pentose phosphate pathway intermediates in PDAC tumour tissues from *Kras*$^{G12D/+}$ *Trp53*$^{R172H/+}$ *Atg7*$^{+/+}$ (n=6) and *Kras*$^{G12D/+}$ *Trp53*$^{R172H/+}$ *Atg7*$^{+/-}$ (n=8) animals measured by LC-MS. Mann-Whitney test was used for statistics. ns, not significant.

Supplemental Figure 7.

(a-b) Repeat experiments of scratch-wound invasion assays showing that *Kras*$^{G12D/+}$ *Trp53*$^{R172H/+}$ *Atg7*$^{+/-}$ cells invade less effectively than *Kras*$^{G12D/+}$ *Trp53*$^{R172H/+}$ *Atg7*$^{+/+}$

counterparts and ATG7 re-expression restores invasive potential of *Kras*$^{G12D/+}$ *Trp53*$^{R172H/+}$ *Atg7*$^{+/-}$ cells.

Supplemental Table 1.

Male/female ratios and overall median survival for all cohorts in the study.

Supplemental Table 2.

Male/female ratios and PDAC-free median survival for all cohorts in the study.

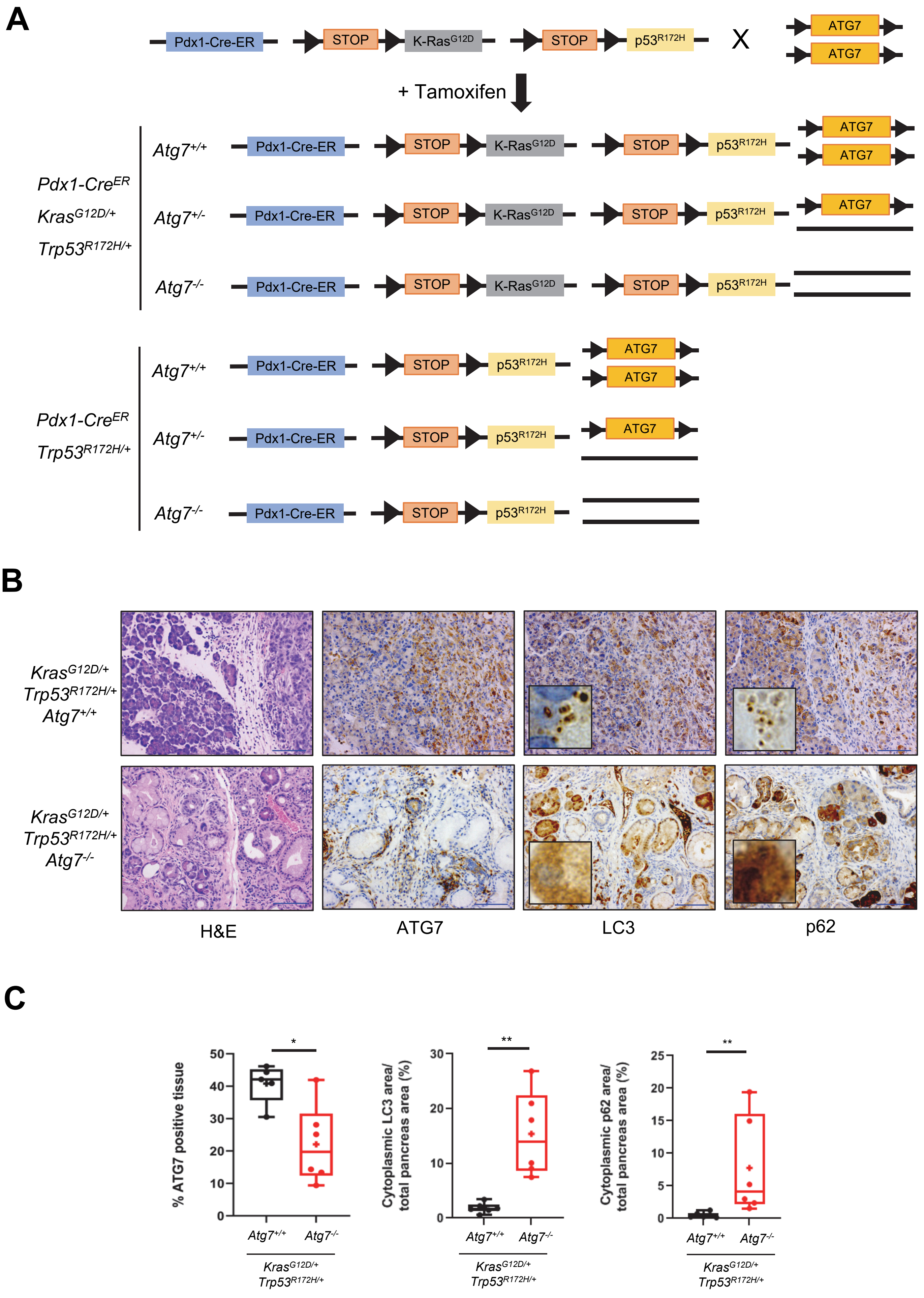

Long et al. Supplementary Figure 1

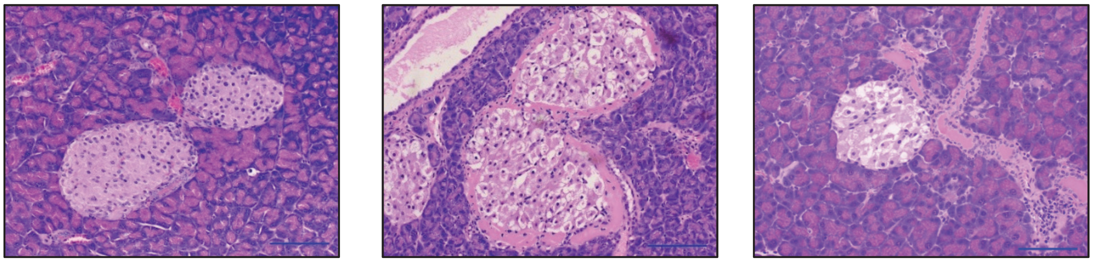

Kras$^{G12D/+}$
Trp53$^{R172H/+}$
Atg7$^{+/+}$

Kras$^{G12D/+}$
Trp53$^{R172H/+}$
Atg7$^{-/-}$

Trp53$^{R172H/+}$
Atg7$^{-/-}$

Long et al. Supplementary Figure 2

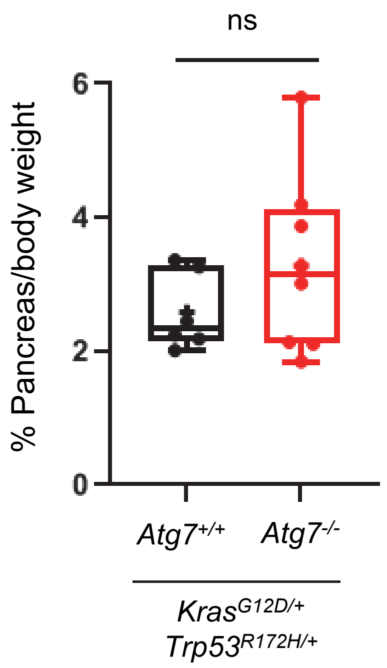

Long et al. Supplementary Figure 3

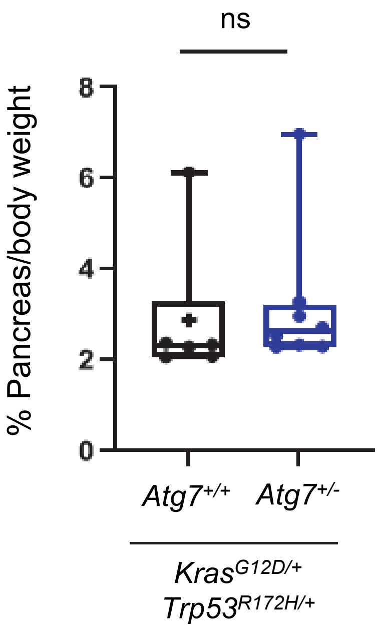

Long et al. Supplementary Figure 4

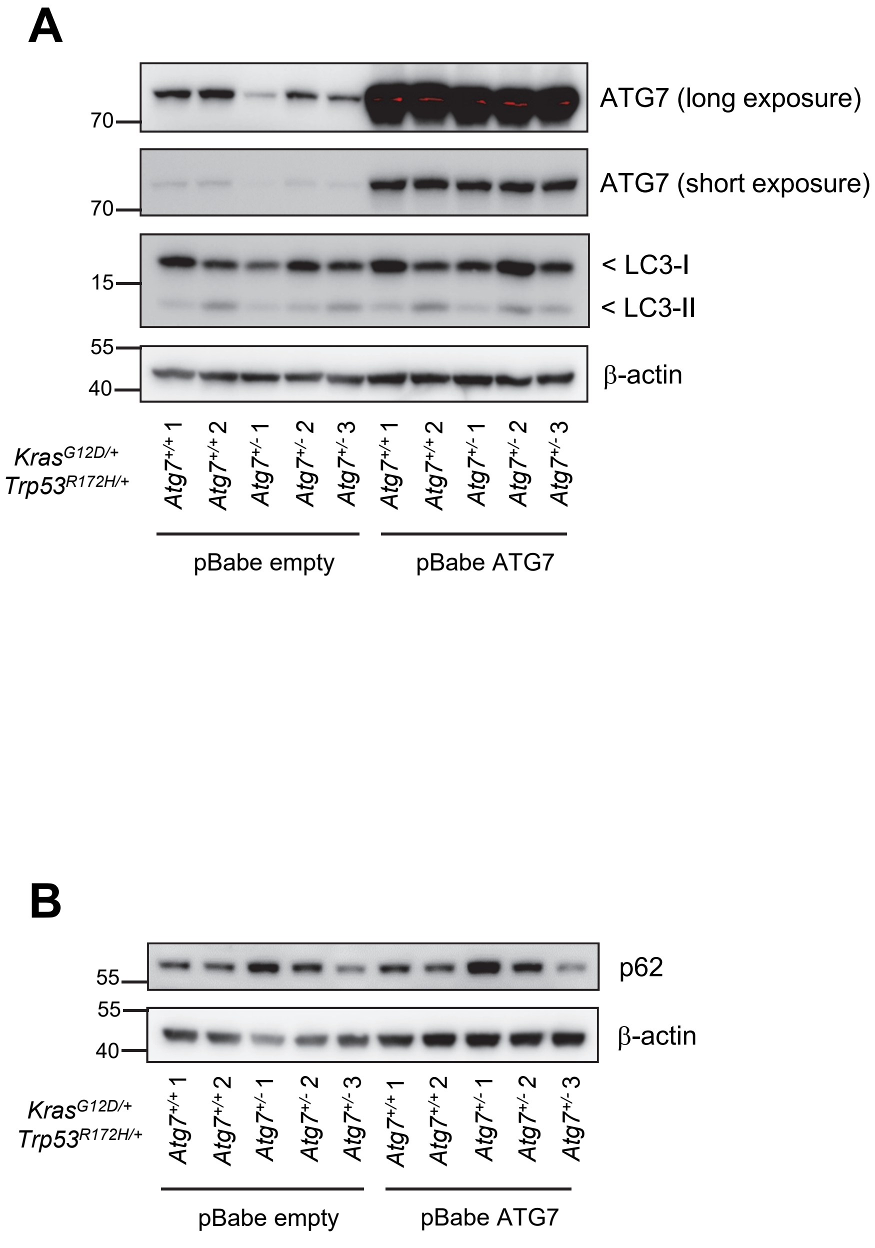

Long et al. Supplementary Figure 5

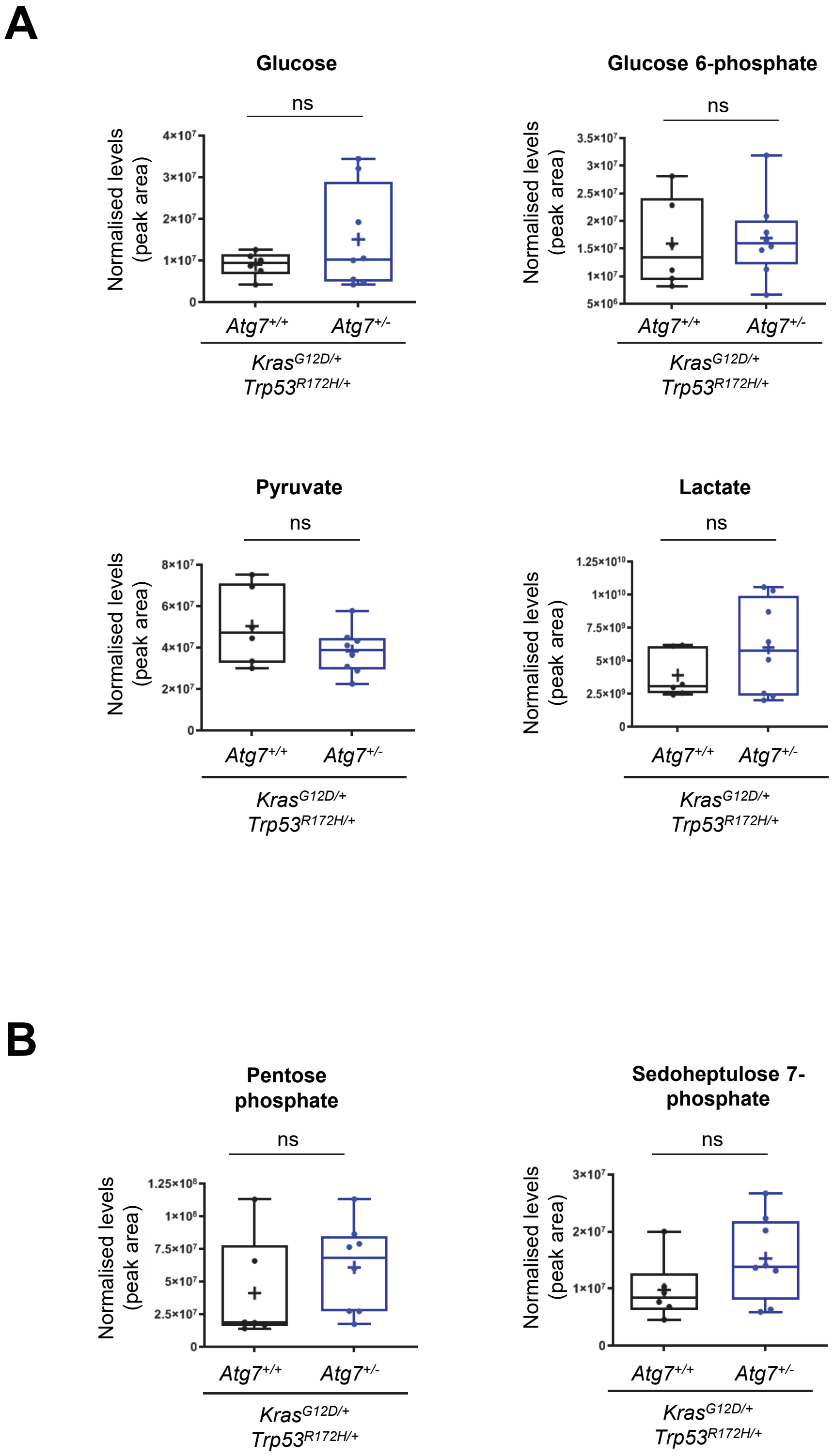

Long et al. Supplementary Figure 6

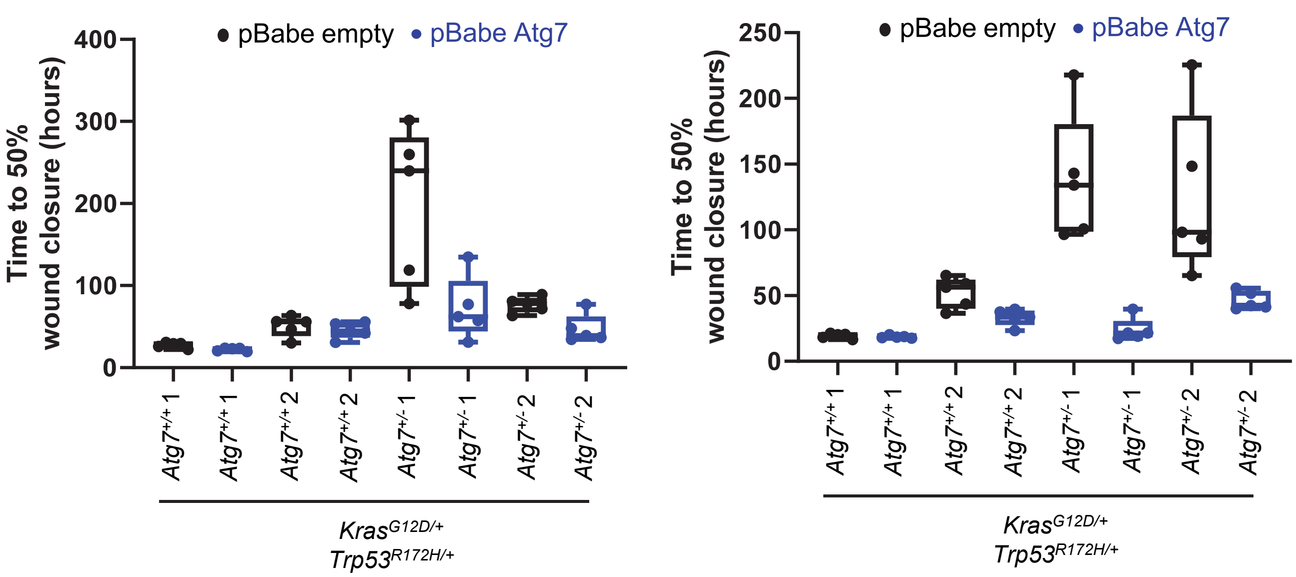

Long et al. Supplementary Figure 7

Cohorts	Male/ female	Overall median survival (days)
$Kras^{G12D/+}$ $p53^{172H/+}$ $ATG7^{+/+}$	10/17	251
$Kras^{G12D/+}$ $p53^{172H/+}$ $ATG7^{+/-}$	11/15	249
$Kras^{G12D/+}$ $p53^{172H/+}$ $ATG7^{-/-}$	25/12	114
$p53^{172H/+}$ $ATG7^{+/+}$	3/4	384
$p53^{172H/+}$ $ATG7^{+/-}$	5/4	479
$p53^{172H/+}$ $ATG7^{-/-}$	13/5	123

Cohorts	Male/ female	PDAC median survival (days)
$Kras^{G12D/+}$ $p53^{172H/+}$ $ATG7^{+/+}$	10/14	260
$Kras^{G12D/+}$ $p53^{172H/+}$ $ATG7^{+/-}$	11/13	253
$Kras^{G12D/+}$ $p53^{172H/+}$ $ATG7^{-/-}$	3/9	141
$p53^{172H/+}$ $ATG7^{+/+}$	0	0
$p53^{172H/+}$ $ATG7^{+/-}$	0	0
$p53^{172H/+}$ $ATG7^{-/-}$	0	0
